# Dietary grape seed extract mitigated growth retardation, hormonal delay, and gastrointestinal toxicity induced by insecticide imidacloprid in Nile tilapia

**DOI:** 10.1007/s10695-025-01475-1

**Published:** 2025-03-25

**Authors:** Nadia A. El-Fahla, Heba M. A. Abdelrazek, Alyaa S. Fouad, Seham A. Helmy, Mohamed T. A. Soliman, Lobna A. Badawy, Nahla S. El-Shenawy

**Affiliations:** 1 Department of Zoology, Faculty of Sciences, Suez Canal University, Ismailia, 41522 Egypt; 2 Department of Physiology, Faculty of Veterinary Medicine, Suez Canal University, Ismailia, Egypt; 3 Department of Cytology and Histology, Faculty of Veterinary Medicine, Suez Canal University, Ismailia, Egypt; 4 Department of Medical Laboratory Sciences, College of Applied Medical Sciences, University of Bisha, 67614 Bisha, Saudi Arabia; 5 Department of Fish Resources and Aquaculture, Faculty of Environmental Agriculture Sciences, Arish University, El-Arish, Egypt

**Keywords:** Digestive enzymes, Grape seed extract, Histopathology, Hormones, Imidacloprid

## Abstract

Overusing the insecticide imidacloprid (IMD) in agriculture has led to its presence in water bodies, causing serious environmental issues and fish toxicity. This study explored the potential benefits of grape seed extract (GSE) in mitigating IMD-induced growth and hormonal and gastrointestinal toxicity in Nile tilapia. A total of 240 healthy juvenile tilapias *Oreochromis niloticus* (*O. niloticus*), weighing an average of 11.44 ± 2.01 g at 2 weeks of age, were divided equally into four groups, each with three replicates. The control group received no special treatment, while the second group was given a diet containing 2% GSE^®^. The third group was exposed to 1.5 µg IMD per liter of water. The fourth group was subjected to the same IMD exposure and fed a diet containing 2% GSE^®^. These treatments were administered continuously for 75 days. Growth indices, survival rate, biochemical parameters, and digestive enzymes were measured. In addition, the growth-related hormones, intestinal malondialdehyde (MDA), and catalase (CAT) were evaluated. Histological evaluations were conducted on the stomach, duodenum, ileum, and hepatopancreas, alongside body composition analysis. Exposure to IMD delayed growth, impaired serum biochemistry and digestive enzyme activity, altered body composition, obstructed hormonal responses, decreased CAT activity, and increased intestinal MDA. All tissues exhibited signs of degradation. Combining dietary GSE^®^ with IMD improved the former parameters affected by IMD. In conclusion, research suggests that incorporating GSE^®^ into the diet may help reduce the adverse effects of IMD exposure on Nile tilapia, presenting a promising opportunity to address the environmental impacts of insecticide contamination in aquatic ecosystems.

**Graphical Abstract:**

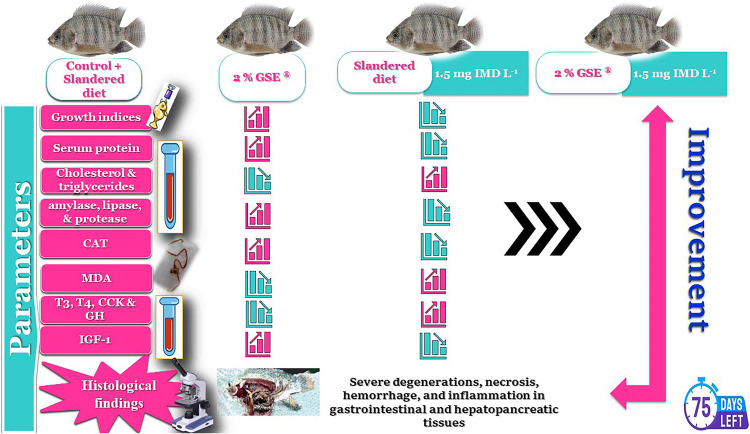

**Supplementary Information:**

The online version contains supplementary material available at 10.1007/s10695-025-01475-1.

## Introduction

Pesticide applications in crop agriculture are increasing to keep up the constant growth of food production to meet human demands and defend plants against foreign organisms (Abdel-Tawwab et al. 2021a). Agrochemical pesticides, including neonicotinoid insecticides, are essential for safeguarding crops and sustaining profits worldwide, but their growing use also has detrimental effects on the environment and public health (Tudi et al. 2021; Miraglia et al. 2009). Minor quantities of pesticides prove efficient in pest management, with the surplus being released into the environment, particularly the aquatic ecosystem, where they are absorbed by marine organisms such as fish, resulting in adverse effects (Abdel Rahman et al. 2023). Eventually, they find their way into the food chain, threatening natural equilibrium and environmental biodiversity, where almost 50% of surface water pollution derives from agricultural sources (Morrissey et al. 2015).

Neonicotinoids are the fastest-growing insecticide due to their potent activity, rain resistance, and flexibility of use on seeds (Abdel-Tawwab et al. 2021a; Simon-Delso et al. 2015). Despite their highly stable chemical characteristics and high solubility in water, neonicotinoids have the potential to enter aquatic environments through diverse pathways (Lu et al. 2024). Imidacloprid (IMD) is one of the most extensively employed neonicotinoid insecticides, favored for its effectiveness in controlling insects and pests, particularly on crops (Abdel Rahman et al. 2023; Lu et al. 2024). It is commonly found in water-based environments, exhibits biodegradation resistance (Todey et al. 2018), and remains stable against hydrolysis at different water pH levels (Tišler et al. 2009; Todey et al. 2018).

According to the study of Lu et al. (2024), crayfish exposed to IMD (88.17 μg /L) revealed a significant decrease in hepatopancreatic and plasma acetylcholine levels. In addition, IMD reduces intestinal flora diversity, increases harmful bacteria, and disrupts microbiota function. Transcriptomic analysis showed a significant rise in differentially expressed genes (DEGs) with higher IMD concentrations, indicating inhibition of neurotransmitter transduction, immune responses, and energy metabolism. Several studies have been conducted on the aquatic ecotoxicity of IMD to determine the lethal IMD doses for aquatic organisms (Rahman et al. 2023; Armbrust and Peeler 2002; Fu et al. 2022; He et al. 2022).

Exposing aquatic organisms to pesticide pollutants can disrupt the balance between exogenous and endogenous reactive oxygen species (ROS) production. This disruption may lead to a decrease in antioxidant defense systems, potentially resulting in complete oxidative damage to the cells (Naiel et al. 2020a). Generally, IMD exposure induces stress leading to the generation of free radicals, causing oxidative damage (Naiel et al. 2020a), which has negative effects on the immune systems (Ewere et al. 2020), cellular antioxidants (Günal et al. 2019), and scavenger enzyme systems (Hong et al. 2020). In addition, several investigations have recorded the various toxicity consequences of IMD in Nile tilapia. These consequences include oxidative stress (Naiel et al. 2020a), gene downregulation, hematological disorders (Américo-Pinheiro et al. 2019), as well as histopathological changes (Ansoar-Rodríguez et al. 2016). Antioxidant enzymes including glutathione peroxidase (GPx), catalase (CAT), and superoxide dismutase (SOD) are the main line of defense against free radicals in animal cells, preventing cellular membrane damage and restoring normal cellular functions (Hong et al. 2024). In marine organisms, these enzymes act as indicators of oxidative stress (Mohammadi et al. 2021b).

Nile tilapia (*Oreochromis niloticus*) is the most commonly farmed fish species globally, accounting for about 5% of global aquaculture production of more than 4.41 million tons in 2022 (Fisheries and Aquaculture 2022). Moreover, fish are natural indicators for detecting direct pesticide accumulations in water, which can indirectly affect humans through the food chain (Ray and Shaju 2023). Using chemical compounds to counteract the toxic effects of pesticides, such as IMD, in aquatic ecosystems can be expensive. IMD can enter water bodies during spraying or from agricultural runoff, and it is confirmed to be highly toxic to marine animals (Nugnes et al. 2023). Fish can absorb IMD through their gills, skin, and digestive system. Biotransformation and detoxification occur in the hepatocytes, which can lead to oxidative damage (Banaee et al. 2024). The reported LC_50_ values for imidacloprid are as follows: 0.13 mg/L for rainbow trout, 1.3 mg/L for common carp, 0.29 mg/L for Nile tilapia, and 0.13 mg/L for fathead minnow (Naiel et al. 2020a, b; Abd El-Hameed et al. 2021b). Additionally, exposure to sub-lethal concentrations of IMD can lead to immune system suppression in *O. niloticus* (Naiel et al. 2020a), changes in blood biochemical parameters in *O. niloticus* (Abdel-Tawwab et al. 2021b), histopathological damage in *Clarias gariepinus* (Abdel Rahman et al. 2022), and oxidative damage in *Cyprinus carpio* (Banaee et al. 2024). However, data on IMD levels specifically within tilapia aquaculture systems remain limited. Therefore, it is crucial to find alternative eco-friendly food additives to mitigate the toxicity caused by IMD (Abdel-Tawwab et al. 2021a).

In aquaculture, plant seeds serve as valuable natural sources of pharmaceutical and bioactive compounds that enhance animal production as well as the health, safety, and quality of animal food; in addition, they are safe as fish food additives (Bai et al. 2015; Bae et al. 2022). Plant seed extracts are eco-friendly products that contain compounds beneficial for aquaculture. Reports indicate they reduce toxicity and stress, prevent diseases, and enhance growth by stimulating appetite and boosting immunity (Abdel Rahman et al. 2020; Tarricone et al. 2023; Xu et al. 2021; Yang et al. 2023). Among these, proanthocyanidins are phenolic compounds found in both edible and non-edible plants, in seeds, fruits, and vegetables (Mattioli and Francioso 2020). They are beneficial food additives for fish health due to their antioxidant properties (Xu et al. 2021; Yang et al. 2023). This safe natural phytochemical is FDA-approved and considered safe for animal feed up to 100 mg/kg, except for dogs (Additives et al. 2016; Li et al. 2020).

Grape seeds (*Vitis vinifera L*) contain 50 mg/kg of proanthocyanins (Zhou et al. 2022), potent antioxidants that surpass vitamins C and E in efficacy (Singh et al. 2023). These compounds promote health by reducing inflammation and enhancing toxin resistance (Shi et al. 2003; Yang et al. 2023). Grape seed extract (GSE) improves antioxidant enzyme activity, growth, and serum parameters in tilapia (Zhai et al. 2014) and rainbow trout (Arslan et al. 2018). Studies show that GSE supplementation enhances fish growth, serum biochemistry, fillet protein content, and liver antioxidant defenses (Mohammadi et al. 2021b; Yang et al. 2023). Additionally, GSE improves tilapia muscle composition, texture, and beneficial fatty acid content, making it a valuable feed additive in aquaculture (Li et al. 2022).

The adverse impacts on aquatic organisms exposed to neonicotinoid insecticide have been reported, but the causal link between IMD exposure and endocrine alterations in fish remains limited. Also, GSE has greater antioxidant stimulatory properties; although, there is limited research on the role of GSE in enhancing fish resistance to pollution. Therefore, supplementing GSE could stand as a novel technique in the aquaculture industry for improving fish growth and health. This study is the first to document the effects of GSE as a dietary supplement on the toxicity of IMD in Nile tilapia. It investigated the potential protective role of GSE against oxidative stress, growth inhibition, body composition changes, biochemical alterations, digestive enzyme suppression, hormonal delays, and gastrointestinal toxicity due to IMD exposure. It also examined the histological changes in the stomach, duodenum, ileum, and hepatopancreas resulting from IMD exposure and the possible supportive role of GSE.

## Materials and methods

### Experimental tilapia

After the 2-week acclimatization period, 240 healthy juvenile tilapia *O. niloticus* with an initial weight of 11.44 ± 2.01 g 2 weeks’ age were used. They were purchased from the Fish Research Center, Suez Canal University, Ismailia governorate, Egypt, and transported to the laboratory. The fish were distributed in a ratio of 20 fish per 60 L aerated glass aquarium (60 × 30 × 40 cm^3^), three aquaria/replicate. Water quality was monitored weekly in each aquarium using a multiparameter photometer (Model HI83308-01, HANNA Instruments, Canada). pH levels ranged from 7.1 to 7.5, alkalinity from 70 to 80 mg/L, water hardness from 350 to 400 mg/L, and ammonia levels from 0.05 to 0.2 mg/L. Dissolved oxygen was kept between 6.2 and 8.1 mg/L using air pumps, while free chlorine levels ranged from 0.1 to 0.2 mg/L. The feces and the food remnant were siphoned after 2 h of feeding. The water temperature was regulated within the 26–28 °C range through submerged heaters. A 12-h light/12-h dark photoperiod was implemented. A static/renewal bioassay method was employed. Aquariums were cleaned three times a week by replacing half of the water and adjusting the tested chemical concentration as necessary. Only healthy, disease-free fish were used for experimentation, and their initial body weight (WI) and length (TLI) were recorded on day zero.

### Experimental design and diets

The fish were randomly allocated into four equivalent groups (three replicates for each group), with 20 fish/aquarium in each replication, as described in Table S1. Control was the first group that fed on a standard diet (0% GSE^®^). A diet supplemented with 2% GSE^®^ (95% purity, red powder form, Hangzhou Joymore Technology Co., China) was added to the second group (Mohammadi et al. 2021b). The third group received a standard diet and was exposed to 1.5 µg/L IMD (Confidor, 70% IMD, Bayer Crop Science, Germany) in water. The fourth group was given 2% GSE^®^ and exposed to the same IMD concentration of 1.5 µg/L in water. The latter dose was selected based on analysis of IMD residue in Suez Canal area aquafarms and comparable matched to the concentration used in Abdel Rahman et al. (2023) study. Also, the chosen concentrations reflect worst-case scenarios or typical exposure levels. The water quality and diet composition were the same for all experimental groups. The only variables were the dietary GSE^®^ and IMD in water.

The dietary composition is detailed in Table S2. GSE^®^, comprising 95% proanthocyanidin, was blended with the food components to create experimental diets at concentrations of 0 (standard diet) and 200 mg/kg (supplemented diet), established based on prior research findings (Jahanbakhshi et al. 2023; Mohammadi et al. 2021a). The basal diet was designed to provide 37.75% crude protein (CP), 6.06% lipid, and 19.65 MJ/kg gross energy (NRC, 2011). The pellets were manufactured using a California pelleting machine with a 2-mm diameter at the Fish Research Center, Suez Canal University, Ismailia, Egypt. The diets were prepared weekly to maintain freshness and prevent spoilage and stored in clean plastic boxes within the refrigerator. The treatment lasted 75 days, and the experimental design was sanctioned by the Scientific Research Ethics Committee, Faculty of Science, Port Said University (ERN: PSU.Sci.50).

### Growth performance and survival rate

At the end of the experiment, the fish’s final body weight (WF) and final total length (TLF) were recorded to determine weight gain (WG), total length gain (TLG), specific growth rate (SGR), and protein efficiency ratio (PER). The fish growth parameters and feed utilization were assessed using the equations outlined by Doan et al. (2017):

$$\mathbf W\mathbf e\mathbf i\mathbf g\mathbf h\mathbf t\boldsymbol\;\mathbf g\mathbf a\mathbf i\mathbf n\left(\mathbf W\mathbf G\right)=\text{average final weight}\left(\text{g}\right)-\text{average initial weight}(\text{g})$$

$$\mathbf S\mathbf p\mathbf e\mathbf c\mathbf i\mathbf f\mathbf i\mathbf c\boldsymbol\;\mathbf g\mathbf r\mathbf o\mathbf w\mathbf t\mathbf h\boldsymbol\;\mathbf r\mathbf a\mathbf t\mathbf e\boldsymbol\;(\mathbf S\mathbf G\mathbf R)=\lbrack(\text{Ln}.\text{average WF}-\text{Ln}.\text{average WI})\text{x}100\rbrack/\text{experimental periods}\;(75\text{days})$$

$$\mathbf P\mathbf r\mathbf o\mathbf t\mathbf e\mathbf i\mathbf n\boldsymbol\;\mathbf e\mathbf f\mathbf f\mathbf i\mathbf c\mathbf i\mathbf e\mathbf n\mathbf c\mathbf y\boldsymbol\;\mathbf r\mathbf a\mathbf t\mathbf i\mathbf o\boldsymbol\;(\mathbf P\mathbf E\mathbf R)=\text{WG}(\text{g})/\text{protein intake}(\text{g})$$

$$\mathbf T\mathbf o\mathbf t\mathbf a\mathbf l\boldsymbol\;\mathbf l\mathbf e\mathbf n\mathbf g\mathbf t\mathbf h\boldsymbol\;\mathbf g\mathbf a\mathbf i\mathbf n\left(\mathbf T\mathbf L\mathbf G\right)=\text{average final length}\left(\text{cm}\right)-\text{average initial length}\left(\text{cm}\right)$$

The aid of the following formula determined the experimental fish survival rate:

$$\text{Survival rate}\;(\text{SR})(\%)=\lbrack\text{Number of fish}\;(\text{after}75\text{days})/\text{Number of fish}\;(\text{initial})\rbrack\times100$$

### Fish sampling

Before the end of the experiment, all fish underwent a 24-h fasting period. Blood samples were taken from the heart of each surviving fish, using clove oil/ethanol (1:10) as anesthesia, following the procedure of Van Doan et al. (2020). Six blood samples per group were gathered in plain test tubes, allowed to clot, and subsequently centrifuged at 3000 rpm for 15 min at room temperature (20–25 °C). The separated sera were stored at − 80 °C to determine biochemical parameters (total protein, albumin, globulin, cholesterol, and triglycerides) and digestive enzymes (α-amylase, lipase, and protease) as well as growth-related hormones: triiodothyronine (T3), thyroxine (T4), insulin growth factor 1 (IGF-1), cholecystokinin (CCK), and growth hormones (GH). Six intestines per group were stored at − 80 °C to analyze catalase (CAT) and lipid peroxidation (MDA).

The cardiac stomach, which includes the glandular and fundic regions, as well as the duodenum, proximal and distal areas of the ileum, and the hepatopancreas (notably, the left lobe of the tilapia liver is a compact organ containing pancreas tissues dispersed within hepatic tissues), was dissected from fish in all treatment groups for histological assessment (Fig. S1). The tissues (six per group) were rinsed in physiological fish saline (0.60% NaCl) and then placed in the Bouin’s solution (comprising 750 mL saturated aqueous picric acid solution, 250 mL 40% formaldehyde, and 50 mL glacial acetic acid), following the method described by Wolf et al. (2004). Subsequently, the tissues were preserved in 70% ethanol. Five fish/groups were stored at − 80 °C for further body composition analysis.

### Serum biochemical parameters

Six blood samples from each group were used to determine the biochemical parameters in sera according to the manufacturer’s protocol for the commercial kits. Total protein concentrations were evaluated using a specific Biuret kit (Catalog No. 41951,41951S), CliniChem Co., USA, at 546 nm, according to Leeman et al. (2018). The albumin levels were determined using a specific Biuret kit (catalog no. 41252,41252S, CliniChem Co., USA), following Garcia Moreira et al. (2018). However, total serum globulin was calculated by deducting total serum albumin from the total serum protein (Coles, 1974). Triglyceride concentration was determined using a specific PAP kit (Catalogue No. 47161,47161S, CliniChem Co., USA) (Chauhan et al. 2014). Cholesterol concentrations were measured with a specific PAP kit (Catalogue No. 47061,47061S, CliniChem Co., USA), according to Mizoguchi et al. (2004).

### Serum enzymatic activity

Sera from six samples/a group was used to measure amylase activity using an amylase-specific ELISA kit (Catalog No. SL0111FI, SunLong Biotech Co., USA), according to Bellino et al. (2010). Lipase activity was measured using a specific ELISA kit (Catalog No. SL0113FI, SunLong Biotech Co., USA) (Basnayake and Ratnam 2015). In addition, protease activity was calculated using a specific ELISA kit assay (Catalog No. AR4011, Boster Co., Pleasanton, CA 94566, USA). The procedures were performed according to Ritota and Manzi (2020).

### Lipid peroxidation and catalase activity

About 0.5 g of each of the six intestinal samples were homogenized (1:9, w/v) with ice-cold 154 mmol/L NaCl solution using a homogenizer (Pro 200, Pro Scientific Inc., USA) and then centrifuged at 3000 g for 15 min at 4 °C. Malondialdehyde (MDA) levels and catalase activity (CAT) were measured in the supernatant spectrophotometrically following the instructions of the kits. MDA was measured using a specific ELISA kit (Catalog No. EK750261, AFG Scientific Co., USA), according to Nisimoto et al. (2010). CAT activity was measured with a specific ELISA kit (Catalog No. SL0028FI), SunLong Co., USA.

### Growth-related hormones

Six serum samples/group were analyzed to determine growth-related hormone levels. Sera were analyzed for T3 using a specific ELISA kit (Catalog No. CSB-E08488f, CUSABIO Co., USA). Serum T4 concentrations were estimated using a specific ELISA kit (Catalog No. CSB-E08489f, CUSABIO Co., USA), according to Leonards and Davoren (2006). For the determination of GH, the sera were subjected to a specific ELISA kit (Catalog No. CSB-E12121Fh, CUSABIO Co., USA), according to Bidlingmaier and Freda (2010). Fish IGF-1 was estimated using a specific ELISA kit (Catalog No. CSB-E12122Fh, CUSABIO Co., USA), following the method of Rajpathak et al. (2009). The CCK level was calculated using a specific assay kit (Catalog No. CSB-E13054Fh, CUSABIO Co., USA), according to Rehfeld (2020). All hormone tests were conducted following the manufacturer’s instructions.

### Chemical analysis of whole fish body composition

Fish samples were weighed, cleaned with distilled water, filleted, and dried in an oven at 50 °C. Five fish per group were analyzed for moisture, crude lipids, crude protein (*N* × 6.25), and ash content, following AOAC (2005) standard protocols (Al-Mentafji 2016). All parameters were measured in triplicates to ensure statistical reliability, and the results were monitored and matched to preexisting calibration standards in the laboratory.

To determine moisture content, the initial weight of the samples was recorded before drying. The samples were dried in an oven at 105 °C for 8 to 10 h until a constant weight was achieved. After cooling in a desiccator, the samples were weighed again, and the percentage of moisture content was calculated using the following equation: moisture (%) = (weight after drying (g)/initial weight of the sample (g)) × 100. Crude lipids were estimated using the Soxhlet extraction method with ethyl ether. After assessing moisture content, samples were dried, finely ground, and mixed with ethyl ether for 16 h. The solvent was then evaporated in a water bath, and the weight of the extracted lipids was recorded via the following equation: crude lipid content (%) = (weight of extract (g)/weight of sample (g)) × 100.

The protein content of the fish was determined using the micro-Kjeldahl method with a Kjeltec autoanalyzer (model 1030; Tecator, Häganäs, Sweden). This method involves digesting the sample with concentrated sulfuric acid to convert organic nitrogen into ammonium sulfate followed by dilution, alkalinization with sodium hydroxide, and distillation to release ammonia. The ammonia is collected in boric acid and measured through titration. A factor of 6.25 was applied to convert the total nitrogen content into the crude protein of the fish sample. The ash content of a sample is the residue left after burning samples in a muffle furnace at 550–660 °C until the residue becomes white. The percent of ash was calculated as follows:

$$\text{Percentage}\;(\%)\;\text{of ash}=(\text{weight of ash}\;(\text{g})/\text{weight of sample}\;(\text{g}))\times100$$

### Histological examination

Tissues from the treated and control groups were dehydrated using increasing concentrations of ethanol, cleared with two changes of xylene, and infiltrated with soft paraffin wax, followed by hard paraffin wax. From each treatment group, six tissue samples were sectioned at a thickness of 5 μm using a rotary microtome. The sections were stained with Mayer’s hematoxylin and counterstained with eosin (Avwioro 2011), then examined for pathologic changes using a light microscope. Six random fields/a slide at a magnification of 200 × were investigated using the free Image J software for histomorphometry. The slides of the cardiac stomach’s glandular and fundic regions measure the glandular glands’ length in the mucosa tunic from the base of the glandular tube at the start of the mucosa to the apical part of the tube. In addition, the length and width of the intestinal villi were measured for intestinal histomorphometry. The height was determined by measuring the distance from the base to the apex of each villus. The width was measured at the midpoint of each villus, spanning the two epithelial layers and including the lamina propria. The goblet cells were counted.

### Statistical analysis

The data of individual replicates were pooled and then analyzed for each group. All data were analyzed using the GraphPad Prism software version 8. The obtained data were assessed for normality using the Shapiro–Wilk test and homogeneity of variances using the Bartlett’s test. Then, one-way ANOVA was conducted to identify differences between the groups. Post hoc comparisons were performed using Tukey’s test to explore which group was different from another. The results of these comparisons are presented as the mean ± standard error (SE) and are indicated by different superscript letters (a, b, c, and d). Groups with different letters are considered significantly different at a significance level of *p* < 0.05, where letter “a” represents the highest significant value. In contrast, means that share the same superscript were not significantly different.

## Results

### The growth indices and survival rate

At the beginning of the experimental phase (day zero), all groups had no variations in the WI and TLI values. However, according to Table 1, the group supplemented with GSE^®^ exhibited a significant increase (*p* < 0.05) in WF and TLF compared to the control group. Conversely, the group exposed to IMD showed decreased WF and TLF values compared to the control group. Supplementing the fish exposed to IMD with GSE^®^ significantly increased WF and TLF (*p* < 0.05) compared to those exposed only to IMD.

**Table 1:** Effect of dietary 2% GSE^®^as diet supplement on fish growth indices and survival rate

Treatment	Control	2% GSE^®^	1.5 µg IMD L^−1^	1.5 µg IMD L^−1^ + 2% GSE^®^
Parameters				
WI (g)	**11.9 ± 0.35**	**11.94 ± 0.39**	**11.84 ± 0.52**	**11.86 ± 0.51**
WF (g)	**45.84 ± 2.59 **^**b**^	**56.87 ± 3.21 **^**a**^	**33.91 ± 2.40 **^**c**^	**44.06 ± 2.88 **^**b**^
WG (g)	**33.94 ± 2.58 **^**b**^	**44.93 ± 3.36 **^**a**^	**22.07 ± 2.52 **^**c**^	**32.2 ± 3.4 **^**b**^
SGR	**2.29 ± 0.12 **^**a**^	**2.68 ± 0.14 **^**a**^	**1.78 ± 0.14 **^**b**^	**2.08 ± 0.12 **^**a**^
PER	**0.91 ± 0.07 **^**a**^	**0.95 ± 0.09 **^**a**^	**0.59 ± 0.07 **^**b**^	**0.94 ± 0.08 **^**a**^
TLI (cm)	**7.90 ± 0.25**	**7.88 ± 0.27**	**7.91 ± 0.28**	**7.89 ± 0.26**
TLF (cm)	**13.90 ± 0.33**^**a**^	**13.98 ± 0.39**^**a**^	**12.80 ± 0.38 **^**b**^	**13.9 ± 0.19**^**a**^
TLG (cm)	**6.03 ± 0.41**^**a**^	**6.10 ± 0.50 **^**a**^	**4.89 ± 0.34**^**b**^	**6.01 ± 0.32**^**a**^
SR (%)	**95%**	**98%**	**77%**	**86%**

Data presented as mean ± SE (*n* = 30/group), at *p* < 0.05, according to the statistical test one-way ANOVA. Means of groups with different superscripts (a, b, and c) in the same row indicate that groups were significantly different at *p* < 0.05; in contrast, means that share the same superscript were not significantly different, where **a** represents the highest significant value, while **c** represents the lowest.* WI* initial weight; *WF* final weight; *WG* weight gain = average final weight (g)—average initial weight (g); *SGR* specific growth rate = [(Ln. average W_F_ – Ln. average W_I_) × 100]/experimental periods (75 days); *PER* protein efficiency ratio = WG (g)/protein intake (g); *TLI* initial total length; *TLF* final total length; *TLG* total length gain = average final length (cm)—average initial length(cm); *SR* survival rate = [number of fish (after 75 days)/number of fish (initial)] × 100; *GSE®* grape seed extract; *IMD* imidacloprid

The WG, TLG, SGR, and PER were significantly (*p* < 0.05) boosted in the supplemented group compared to the control one. However, the IMD-exposed group showed significantly (*p* < 0.05) the lowest WG, TLG, SGR, and PER values among the groups. The IMD-exposed group had a lower SR (77%) than the other groups. Providing the GSE^®^ with IMD-exposed tilapia increased the SR value by 86% compared to the IMD-exposed group. The GSE^®^-supplemented group showed an enhancement of SR by 98%.

### Serum protein and lipid profile

The total protein, albumin, and globulin levels in Nile tilapia sera increased significantly in the GSE^®^-supplemented group than in the control fish (Table 2). The later parameters decreased significantly in the IMD-exposed group than control. In IMD-intoxicated fish, the levels of triglycerides and cholesterol were elevated significantly compared to other groups. Adding 2% GSE^®^ to the diet with IMD-intoxicated fish significantly declined the high levels of triglycerides and cholesterol. No significant differences were observed in triglyceride and cholesterol levels between the control fish and the group supplemented with GSE^®^.

**Table 2:** Effect of dietary 2% GSE^®^ as a diet supplement on serum biochemical parameters and digestive enzyme levels, in addition to catalase and lipid peroxidation in the intestine of Nile tilapia after 75 days of the experimental period

Treatment	Control	2% GSE^®^	1.5 µg IMD L^−1^	1.5 µg IMD L^−1^ + 2% GSE^®^
Parameters				
Total protein (g/dL)	**4.07 ± 0.02 **^**b**^	**4.25 ± 0.04**^**a**^	**3.58 ± 0.16 **^**c**^	**3.97 ± 0.03 **^**b**^
Albumin (g/dL)	**2.53 ± 0.01 **^**b**^	**2.68 ± 0.03**^**a**^	**2.23 ± 0.03 **^**d**^	**2.39 ± 0.04 **^**c**^
Globulin (g/dL)	**1.54 ± 0.01 **^**b**^	**1.57 ± 0.02**^**a**^	**1.34 ± 0.15 **^**c**^	**1.53 ± 0.01 **^**b**^
Triglyceride (mg\dL)	**114.60 ± 0.03 **^**c**^	**110.88 ± 1.45**^**c**^	**162.40 ± 1.97 **^**a**^	**132.86 ± 1.87 **^**b**^
Cholesterol (mg\dL)	**91.39 ± 0.01 **^**c**^	**88.81 ± 0.87**^**c**^	**138.79 ± 1.37 **^**a**^	**109.23 ± 1.42 **^**b**^
α-amylase (ng/mL)	**17.93 ± 0.07 **^**b**^	**24.01 ± 0.2**^**a**^	**14.75 ± 0.03 **^**c**^	**18.01 ± 0.19 **^**b**^
Lipase (ng/mL)	**5.20 ± 0.11 **^**b**^	**7.32 ± 0.23**^**a**^	**3.57 ± 0.02 **^**c**^	**5.43 ± 0.13 **^**b**^
Protease (ng/mL)	**6.19 ± 0.12 **^**b**^	**7.82 ± 0.08**^**a**^	**5.81 ± 0.02 **^**c**^	**6.50 ± 0.09 **^**b**^
CAT (pg/mg)	**732.53 ± 4.68 **^**b**^	**765.3 ± 6.53**^**a**^	**489.23 ± 7.43 **^**d**^	**660.57 ± 6.09 **^**c**^
MDA (nmol/mg)	**2.15 ± 0.01 **^**c**^	**2.09 ± 0.01**^**c**^	**3.83 ± 0.02 **^**a**^	**2.30 ± 0.08 **^**b**^

Data represented as mean ± SE (*n* = 6/group), at *p* < 0.05, according to the statistical test one-way ANOVA. Means of groups with different superscripts (a, b, c, and d) in the same row indicate that groups were significantly different at *p* < 0.05; in contrast, means that share the same superscript were not significantly different, where a represents the highest significant value, while d represents the lowest. *CAT* catalase, *MDA* malondialdehyde, *GSE®* grape seed extract, *IMD* imidacloprid

### Serum digestive enzyme activity

The levels of digestive enzymes, including amylase, lipase, and protease, were significantly higher (*p* < 0.05) in the group of fish fed with GSE^®^ compared to the control group. However, IMD-exposed fish revealed a decrease in these enzyme activities. Supplementation of the GSE^®^ to the diet of IMD-intoxicated fish significantly improved digestive enzyme activities compared to the IMD group (Table 2).

### Catalase activity and MDA levels

Exposure to IMD led to a significant increase (*p* < 0.05) in MDA content while concurrently resulting in a significant decrease (*p* < 0.05) in the CAT activity compared to the other groups. The inclusion of GSE^®^ in the group exposed to IMD resulted in a significant decrease (*p* < 0.05) in MDA content, along with a significant increase (*p* < 0.05) in the CAT activity compared to the IMD-exposed group. There are no differences in the levels of MDA contents between GSE^®^ and control groups. The dietary addition of GSE^®^ exerted a beneficial effect (*p* < 0.05) on antioxidant status, as indicated in Table 2, demonstrated by the elevated CAT activity.

### Growth-related hormones

Providing GSE^®^ to the diet of Nile tilapia significantly (*p* < 0.05) promoted IGF-1 to levels higher than the control group while lowering significantly (*p* < 0.05) the levels of T3, T4, and CCK. Following 75 days of IMD exposure, levels of T3, T4, CCK, and GH in sera of tilapia were significantly (*p* < 0.05) increased. In contrast, the levels of IGF-1 decreased significantly (*p* < 0.05) when compared to the control group. The addition of GSE^®^ to IMD-exposed fish significantly lowered levels of T3, T4, GH, and CCK and promoted significantly (*p* < 0.05) IGF-1 as compared to the IMD group (Table 3).

**Table 3:** Effect of dietary 2% GSE^®^ as a diet supplement on levels of growth-related hormones in sera of Nile tilapia at the end of the experimental period.

Treatment	Control	2% GSE^®^	1.5 µg IMD L^−1^	1.5 µg IMD L^−1^ + 2% GSE^®^
Parameters				
T4 (ng/mL)	**42.24 ± 0.24 **^**a**^	**41.36 ± 0.56 **^**b**^	**43.16 ± 0.11 **^**a**^	**41.91 ± 0.19 **^**b**^
T3 (ng/mL)	**2.48 ± 0.09 **^**b**^	**1.86 ± 0.04 **^**c**^	**2.95 ± 0.06 **^**a**^	**2.44 ± 0.05 **^**b**^
GH (pg/mL)	**828.23 ± 11.7 **^**b**^	**830.00 ± 5.20 **^**b**^	**918.37 ± 3.40 **^**a**^	**797.17 ± 4.60**^**c**^
IGF-1 (pg/mL)	**254.3 ± 3.78 **^**b**^	**308.9 ± 2.29 **^**a**^	**199.43 ± 6.59 **^**c**^	**233.53 ± 11.09 **^**b**^
CCK (pg/mL)	**553.9 ± 21.51**^**b**^	**413.87 ± 2.49 **^**c**^	**789.03 ± 4.50 **^**a**^	**558.73 ± 7.69 **^**b**^

Data represented as mean ± SE (*n* = 6/group) at *p* < 0.05, according to the statistical analysis one-way ANOVA. Means of groups with different superscripts (a, b, c, and d) in the same row indicate that groups were significantly different at *p* < 0.05; in contrast, means that share the same superscript were not significantly different, according to post hoc Tukey’s test, where a represents the highest significant value, while d represents the lowest.* T4* thyroxine, *T3* triiodothyronine, *GH* growth hormone, *IGF-1* fish insulin-like growth factor 1, *CCK* cholecystokinin, *GSE®* grape seed extract, *IMD* imidacloprid

### Whole-body composition

**Fig. 1:**
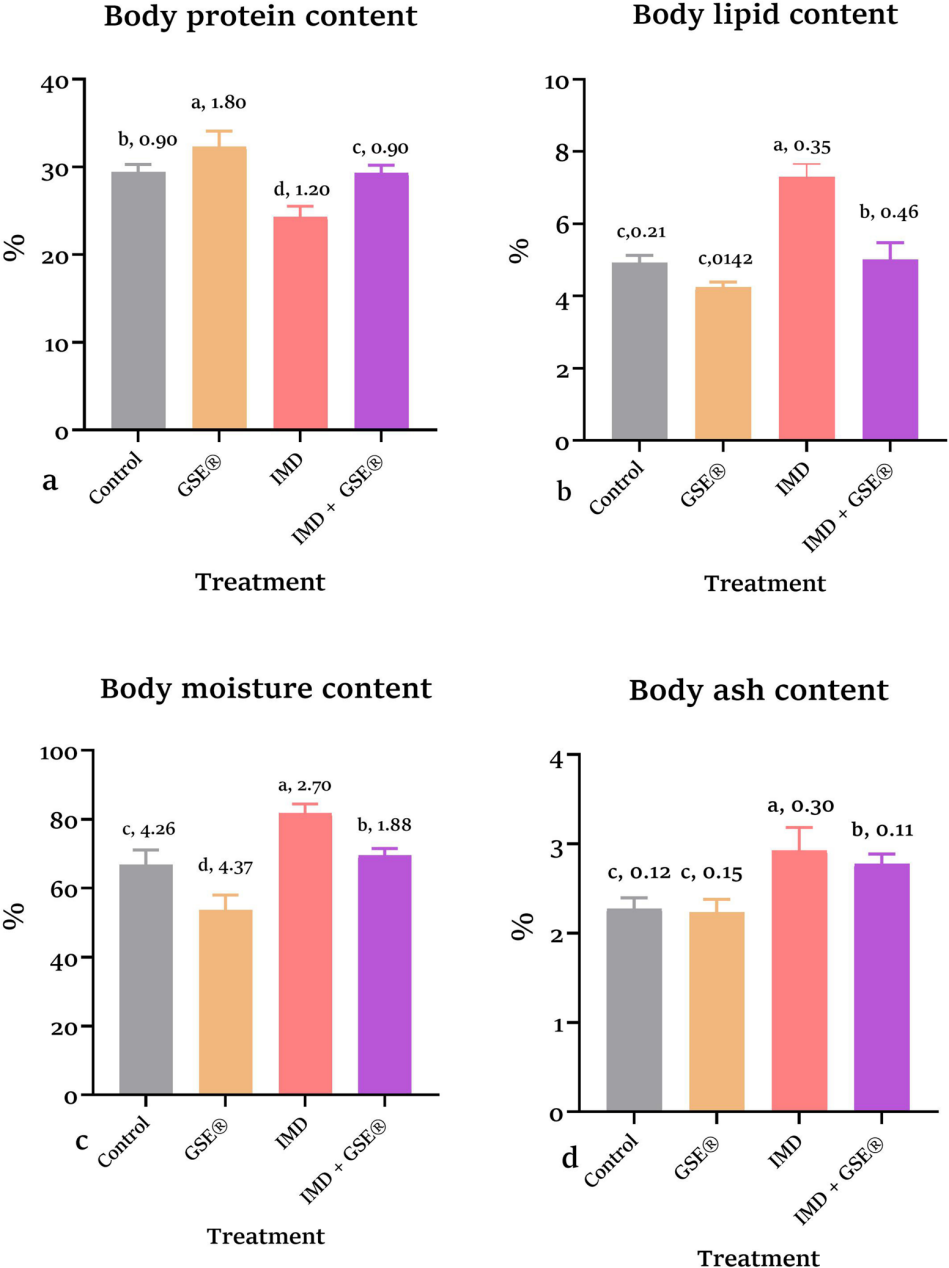
**a, b, c, d** Effect of dietary 2% GSE^®^ on chemical body composition of 1.5 µg IMD L^−1^ intoxicated Nile tilapia after 75 days. Data were expressed as mean ± SE at *p* < 0.05 (*n* = 5/group), according to the statistical test one-way ANOVA. Groups with different letters (a, b, c & d) above bars were significantly different at *p* < 0.05; in contrast, groups that share the same letter were not significantly different, according to post hoc Tukey’s test, where **a** represents the highest significant value and **d** for the lowest. GSE^®^, grape seed extract; IMD, imidacloprid

The body protein content findings indicated that the GSE^®^-supplemented group demonstrated a high mean of body protein of 32.33 ± 1.80%. Still, the group exposed to IMD exhibited a more significant decrease to 24.33 ± 1.20%, both *p* = 0.0007 relative to control (29.34 ± 0.90%). Adding dietary GSE^®^ to IMD-exposed fish significantly (*p* < 0.05) increased the mean protein contents to 29.33 ± 0.90% more than the IMD group. The IMD-exposed group revealed a significant (*p* < 0.05) increase in lipid (7.31 ± 0.35%, *p* = 0.003) (Fig. 1b), moisture (81.81 ± 4.26%, *p* = 0.003) (Fig. 1c), and ashes (2.93 ± 0.30%, *p* = 0.04) contents (Fig. 1d), relative to control. Incorporation of GSE^®^ into the IMD-exposed group significantly decreased lipid (5.01 ± 0.46%), moisture (69.62 ± 1.88%), and ashes (2.78 ± 0.11%) contents relative to control. There was no difference in lipid and ashes content values between the control and GSE^®^ groups.

### Pathological findings

**Fig. 2:**
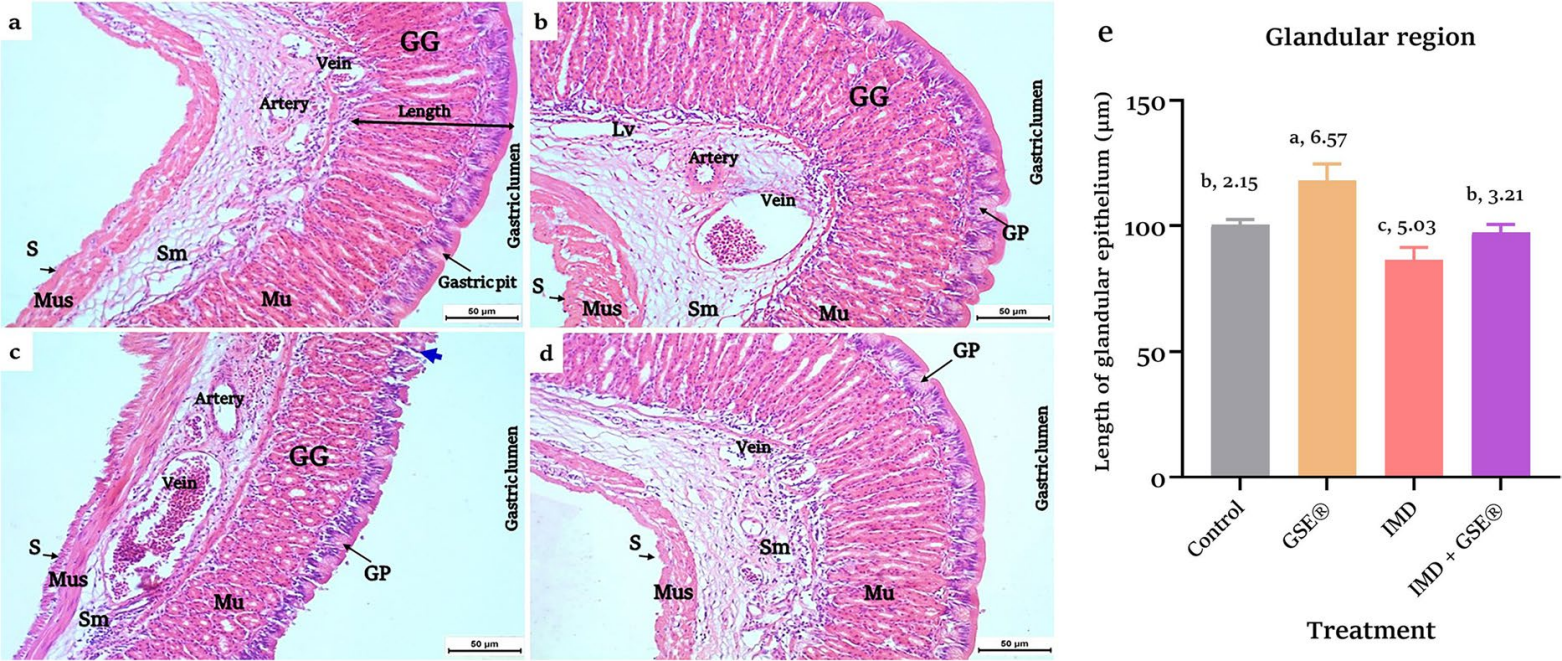
**a, b, c, d, e** Cardiac stomach (glandular region) in control and 2% GSE^®^ supplemented Nile tilapia (**a**) and (**b**), respectively, showed four layers; tunica mucosa (Mu) with well-identified gastric glands (GG) and pits (GP), submucosa (Sm), muscularis (Mus), and the outer layer serosa (S). **c** The IMD-exposed cardiac stomach section (1.5 µg L^−1^) showed dilatation, congestion of gastric veins, and degeneration in gastric pits (blue arrow). **d** Section of IMD-exposed stomach supplemented with 2% GSE^®^ showed a normal appearance of the cardiac stomach. H&E stain (× 200). **e** Morphometric lengths of the gastric epithelium in the cardiac stomach region after 75 days of treatment with 1.5 at *p* < 0.05, according to the statistical test one-way ANOVA. Means of groups with different superscripts (a, b, c, and d) in the same row indicate that groups were significantly different at *p* < 0.05; in contrast, means that share the same superscript were not significantly different, where **a** represents the highest significant value, while **d** represents the lowest, IMD L^−1^ and 2% GSE^®^. Data were expressed as mean ± SE at *p* < 0.05 (*n* = 6/group), according to the statistical test one-way ANOVA. Groups with different letters (a, b, and c) above bars were significantly different at *p* < 0.05; in contrast, groups that share the same letter were not significantly different, according to post hoc Tukey’s test, where **a** represents the highest significant value and **c** for the lowest. Lv, lymphatic vessel; GSE^®^, grape seed extract; IMD, imidacloprid

**Fig. 3:**
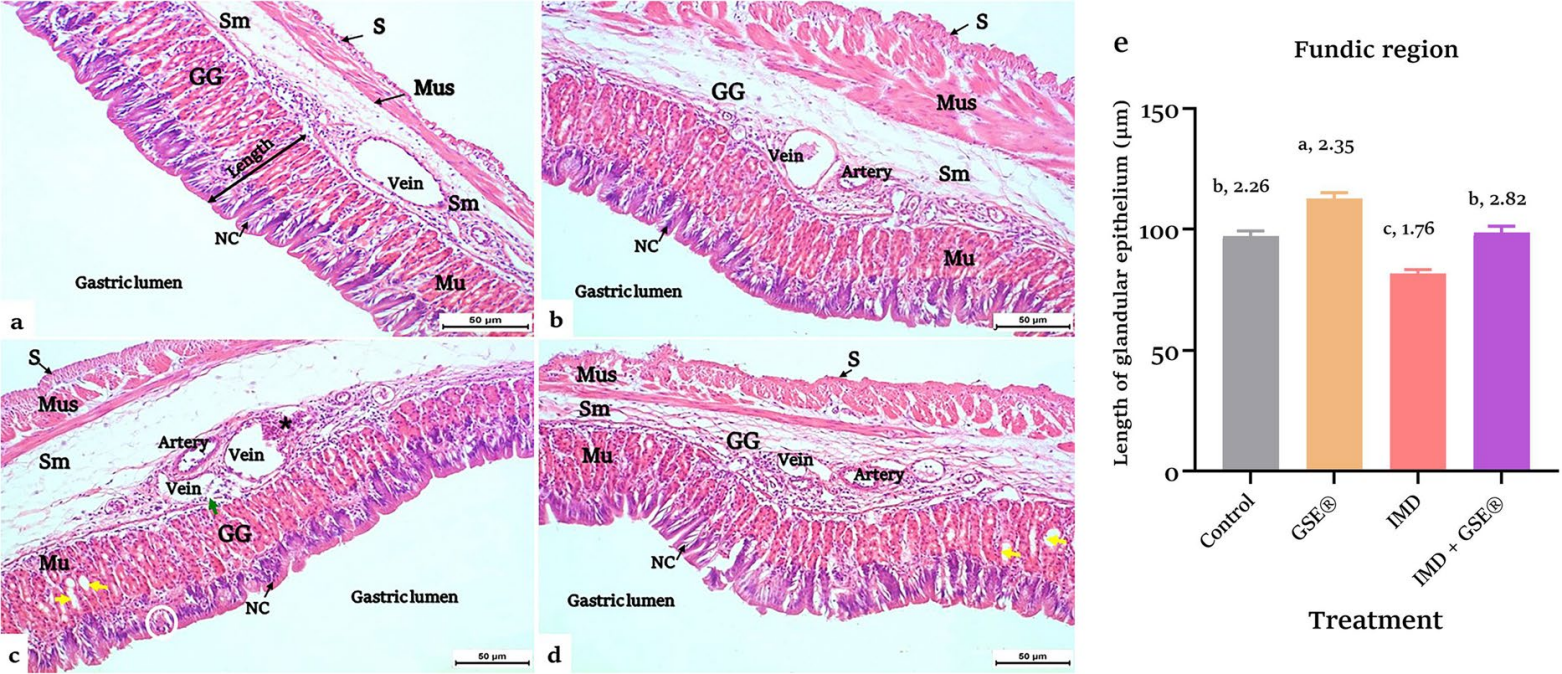
**a, b, c, d, e** Fundic region of the cardiac stomach in control and 2% GSE^®^ supplemented Nile tilapia (**a**) and (**b**), respectively, showed four layers: tunica mucosa filled with gastric glands and covered with columnar cells (Nc), submucosa (Sm), muscularis (Mus), and serosa (S). **c** Funds stomach of 1.5 IMD L^−1^-exposed Nile tilapia showed dilatation of gastric vein, hemorrhage, and infiltration of lymphocytes in the submucosal layer (*), focal necrosis in the neck cell (white circle), and detachment and loss of gastric cells adherence (yellow arrows). **d** IMD-exposed tilapia supplemented with 2% GSE^®^ showed a normal appearance of the fundic stomach with a minor detachment of gastric glands. H&E stain (× 200). Straight yellow lines indicate the linear length of mucosal gastric glands.** e** Morphometric lengths of the gastric epithelium in the fundic stomach region after 75 days of treatment with 1.5 µg IMD L^−1^ and 2% GSE^®^. Data were expressed as mean ± SE at *p* < 0.05 (*n* = 6/group), according to the statistical test one-way ANOVA. Groups with different letters (a, b, and c) above bars were significantly different at *p* < 0.05; in contrast, groups that share the same letter were not significantly different, according to post hoc Tukey’s test, where **a** represents the highest significant value and **c** for the lowest. GSE^®^, grape seed extract; IMD, imidacloprid

The control and GSE^®^ supplemented groups revealed the normal appearance of glandular and fundic regions of the cardiac stomach with no signs of alterations (Fig. 2a, b and 3a and b). Regarding IMD-intoxicated fish, there was dilatation and congestion of gastric veins, degeneration in gastric pits in the glandular stomach (Fig. 2c), hemorrhage and inflammation in the submucosal layer, epithelial necrosis in the neck cell, and loss of gastric adherence in the fundic region (Fig. 3c).

Incorporating dietary GSE^®^ into the group intoxicated with IMD enhanced the histological appearance of gut tissues, resembling that of the control group (Figs. 2d and 3d). After 75 days, the supplemented group revealed a significant increase in the length of gastric glands in gut tissues compared to the control group (Figs. 2e and 3e). However, the gastric mucosal lengths diminished significantly in the IMD-exposed tilapia compared to the control. The addition of GSE^®^ to the IMD-exposed group significantly enlarged the length of the gut glandular layer compared to the IMD group.

**Fig. 4:**
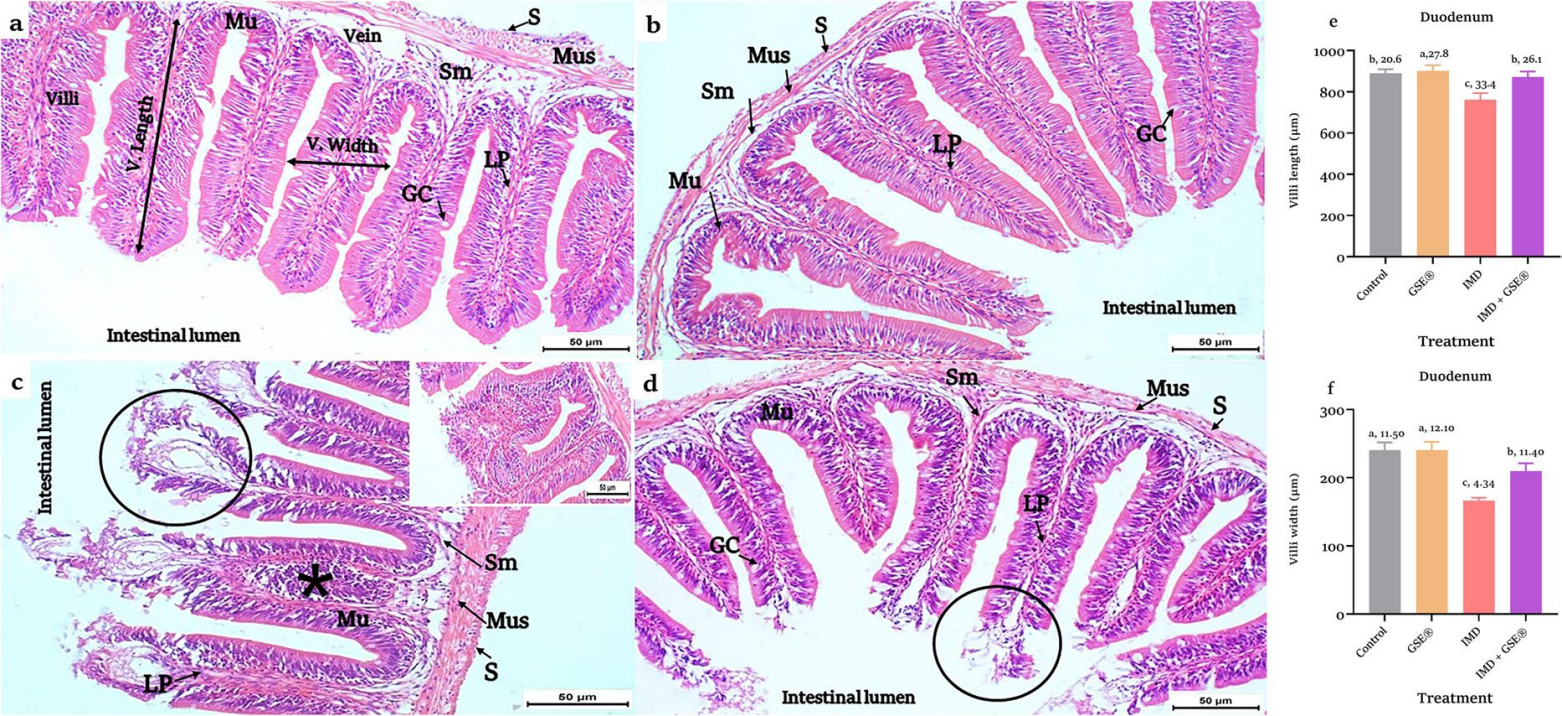
**a, b, c, d, e, f** Duodenal sections in control and 2% GSE^®^ supplemented Nile tilapia (**a**) and (**b**), respectively, showed the four tunics: mucosa (Mu) with mucosal folds (villi) extending into the intestinal lumen, submucosa (Sm), muscular (Mus), and serosa (S). **c** Section of the 1.5 µg IMD L^−1^- exposed (L^−1^) duodenum showed erosion and shedding of the villus epithelium with sloughed debris in the lumen (circles) and mononuclear inflammation (*) in enlarged lamina proparia (LP). **c** The window showed necrosis of intestinal epithelium in two adjacent villi with extensive mononuclear infiltrations and hemorrhage. **d** IMD-exposed tilapia supplemented with 2% GSE^®^ showed a normal appearance of the duodenum besides insignificant sloughing in the apical villi epithelium. H&E stain (× 200). Straight yellow lines indicate the linear measurements of villus length and villus width. **e** and **f** Histomorphometric analysis of villi length and width, respectively, after 75 days of treatment with 1.5 µg IMD L^−1^ and 2% GSE^®^. Data were expressed as mean ± SE at *p* < 0.05 (*n* = 5/group), according to the statistical test one-way ANOVA. Groups with different letters (a, b, and c) above bars were significantly different at *p* < 0.05; in contrast, groups that share the same letter were not significantly different, according to post hoc Tukey’s test, where **a** represents the highest significant value and **c** for the lowest. GC, goblet cell; GSE^®^, grape seed extract; IMD, imidacloprid

**Fig. 5:**
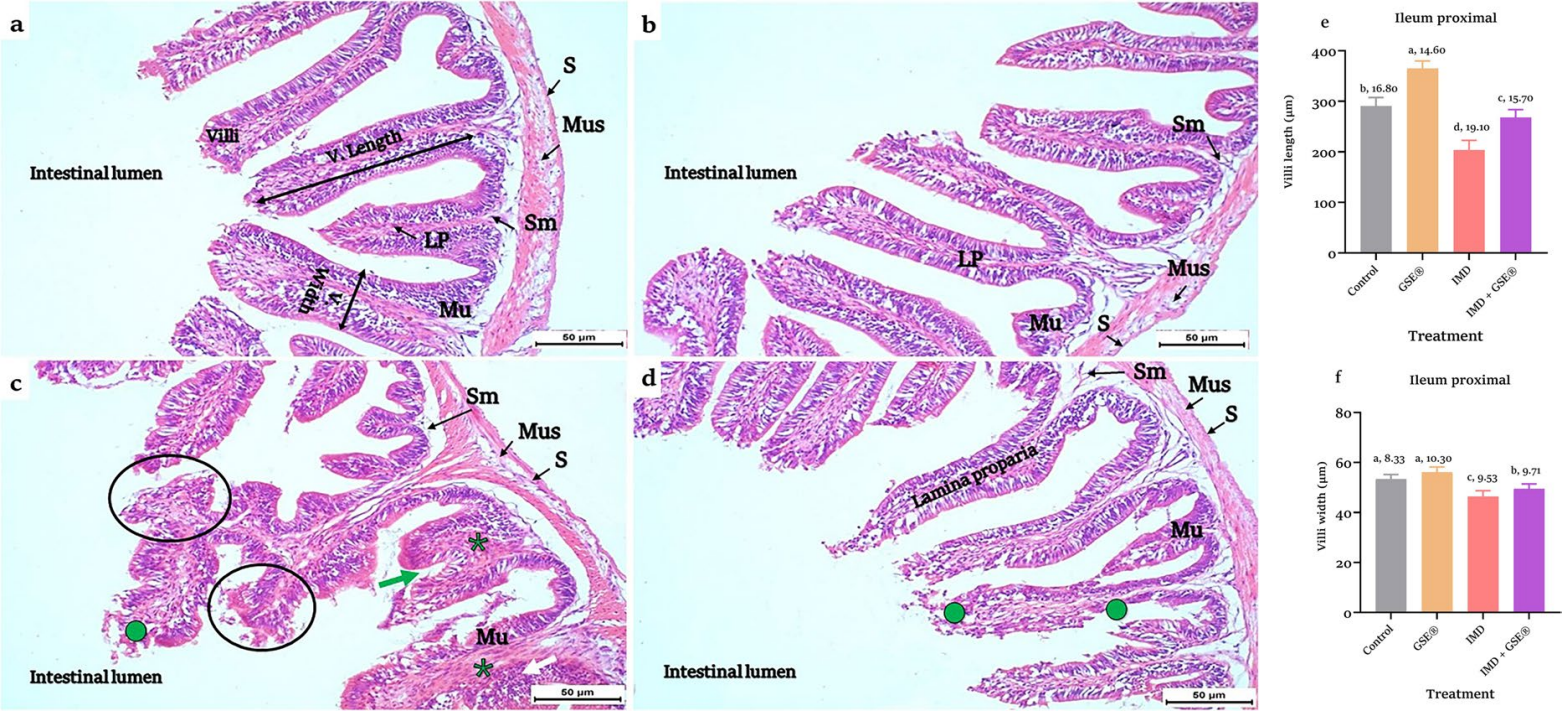
**a, b, c, d, e, f** Sections of the ileum (proximal region) in control and 2% GSE^®^ supplemented Nile tilapia (**a**) and (**b**), respectively, showed the four tunic layers; mucosa (Mu) with branched villi extending into the intestinal lumen, submucosa (Sm), muscularis (Mus), and serosa (S). **c **ileum section of 1.5 µg IMD L^−1^-exposed ileum showed erosion and shedding of the villus epithelium (●) and necrosis of villi epithelium associated with hemorrhage (circles). Degeneration of villi histological structure (green arrow) with mononuclear inflammation (*) in enlarged lamina proparia (LP) was observed. **d** IMD-exposed tilapia supplemented with 2% GSE^®^ showed erosion of villus epithelium (●); however, an improvement in villi histological appearance was detected. H&E stain (× 200). Straight yellow lines indicate the linear measurements of villus length and width. **e** and **f** Histomorphometry of villi length and width, respectively, after 75 days of treatment with 1.5 µg IMD L^−1^ and 2% GSE^®^. Data were expressed as mean ± SE at *p* < 0.05 (*n* = 5/group), according to the statistical test one-way ANOVA. Groups with different letters (a, b, and c) above bars were significantly different at *p* < 0.05; in contrast, groups that share the same letter were not significantly different, according to post hoc Tukey’s test, where **a** represents the highest significant value and **c** for the lowest. GSE^®^, grape seed extract; IMD, imidacloprid

**Fig. 6:**
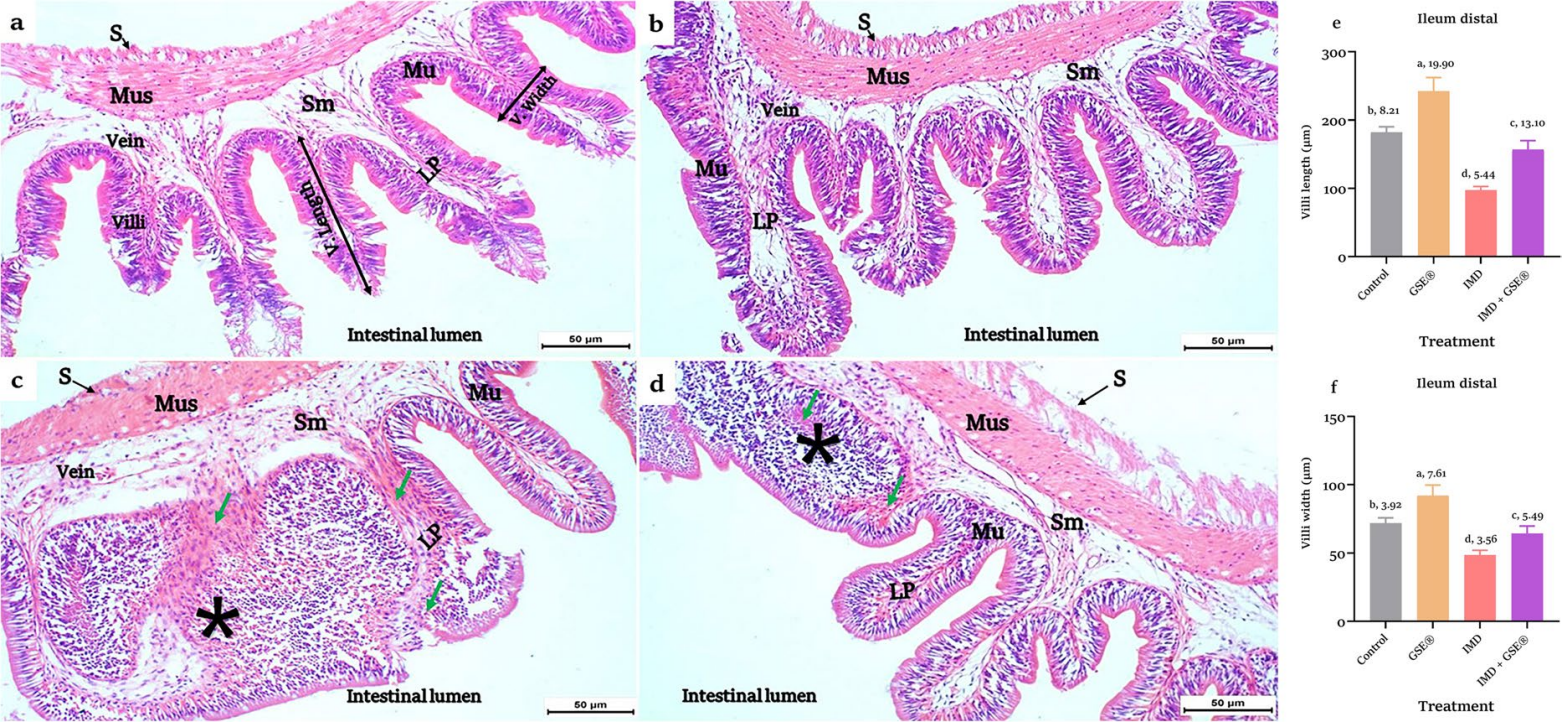
**a, b, c, d, e, f** Sections of the ileum (distal region) in control and 2% GSE^®^ supplemented Nile tilapia (**a**) and (**b**), respectively, showed the four tunic layers: mucosa (Mu) with villi, submucosa (Sm), muscularis (Mus), and serosa (S). **c** Section of 1.5 µg IMD L^−1^-exposed ileum revealed degeneration of villi histological structure, necrosis with mononuclear inflammation (*), and hemorrhage (green arrows). **d** Supplementation of 2% GSE^®^ to IMD-exposed tilapia resulted in the same pathological appearance of IMD-exposed ileum in the distal region. The straight yellow lines represent the linear measurements of villus length and width. **e** and **f** Histomorphometry of villi length and width, respectively, after 75 days of treatment with 1.5 µg IMD L^−1^ and 2% GSE^®^. Data were expressed as mean ± SE at *p* < 0.05 (*n* = 6/group), according to the statistical test one-way ANOVA. Groups with different letters (a, b, and c) above bars were significantly different at *p* < 0.05, according to post hoc Tukey’s test, where **a** represents the highest significant value and **d** for the lowest. LP, lamina propria; GSE^®^, grape seed extract; IMD, imidacloprid

There were no detectable alterations in intestinal architecture, including the duodenum (Fig. 4a and b), proximal (Fig. 5a and b), and distal (Fig. 6a and b) sections of ileum in control and GES^®^ supplemented groups. Most examined sections in IMD-intoxicated tilapia displayed histological degeneration of villi structure, erosion of villi epithelium, and villus necrosis. In addition, there is dilation of lamina proparia, hemorrhage, and mononuclear inflammation (Figs. 4c, 5c, and 6c).

Supplementation of GES^®^ to the diet of IMD-exposed tilapia revealed an improvement in the examined sections of the duodenum and proximal ileum (Figs. 5d and 6d). Conversely, the distal ileum’s tissues exhibit histological alterations (Fig. 6d). Histomorphometry of intestinal villi displayed a significant (*p* < 0.05) increase in the villi length (Figs. 4e, 5e, and 6e) and width (Figs. 4f, 5f, and 6f) of different intestinal regions in fish fed on GES^®^ diet than control and other groups. Also, dietary GES^®^ co-administrated to IMD-exposed tilapia significantly (*p* < 0.05) increased the length and width of villi than the IMD group.

**Fig. 7:**
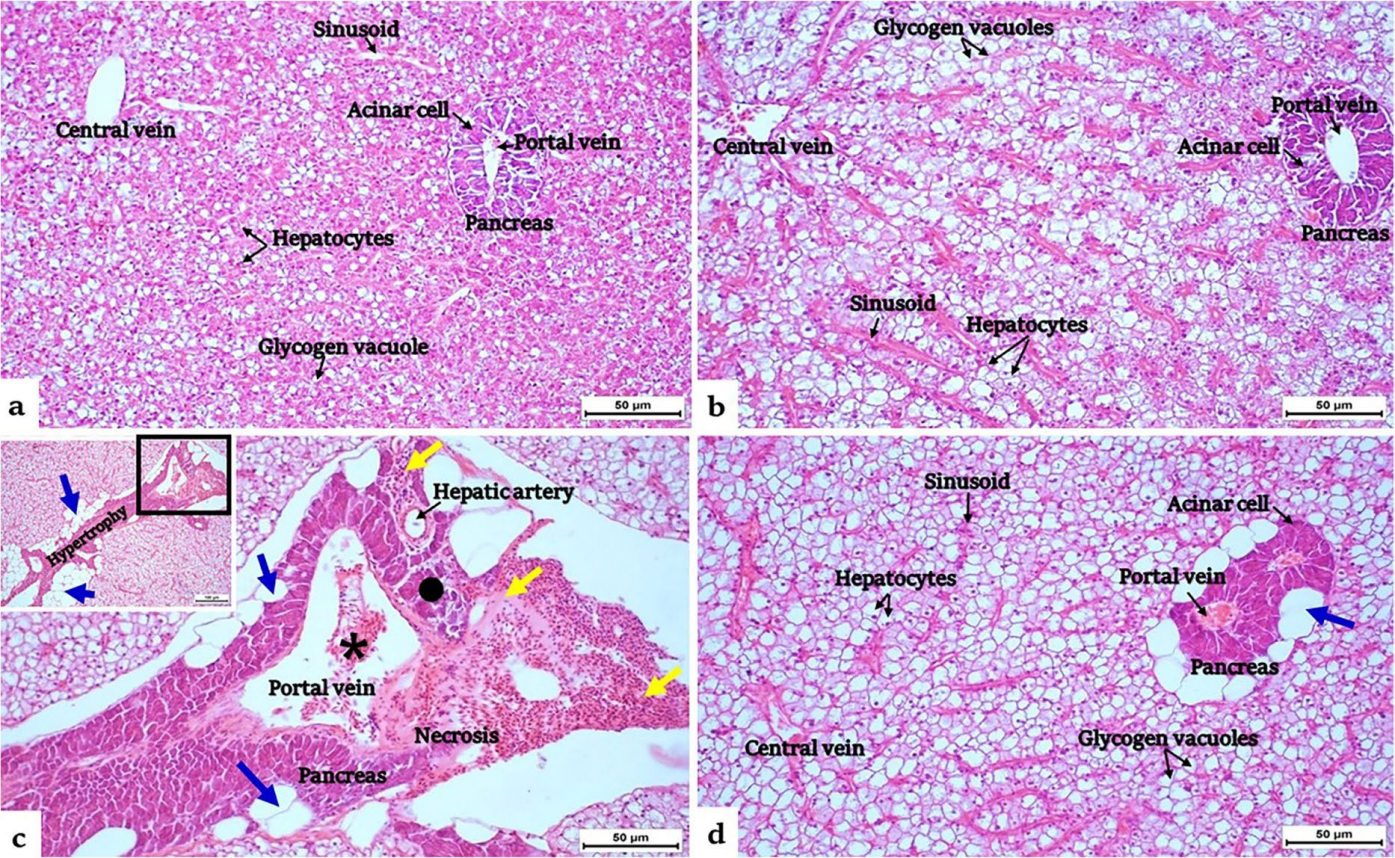
**a, b, c, d** Hepatopancreatic sections in control and 2% GSE^®^ supplemented Nile tilapia (**a) **and (**b**), respectively, showed normal hepatic tissue with polygonal hepatocytes, glycogen vacuoles, and sinusoids dispersed between hepatocytes. Besides, the intrahepatic pancreas with normal acinar cells surrounded by hepatocytes and surrounding the portal vein. **c** Section of 1.5 µg IMD L^−1^-exposed liver showed pancreocytes hypertrophy, degeneration of acinar cell (●), dilation and hemolysis of intravascular hepatic portal vein (*), and hemorrhage with mononuclear aggregations (yellow arrows). In addition, macro-steatosis (blue arrows) was frequently observed. **d** Supplementing 2% GSE^®^ improved the appearance of hepatic and pancreatic tissues in IMD-exposed tilapia, but pancreatic steatosis persisted. **a, b, c, d** H&E stain (× 200) and **c** window, H&E stain (× 100). GSE^®^, grape seed extract; IMD, imidacloprid

The IMD-exposed group showed severe alterations in hepatopancreatic tissues; most examined sections exhibited degenerations in both hepatic and pancreatic tissues. Pancreocyte hypertrophy, degeneration of acinar cells, dilation and hemolysis of intravascular hepatic portal vein, hemorrhage, inflammations, and macro-steatosis were the most detectable lesions (Fig. 7c). Supplementing GSE^®^ improved the appearance of hepatic and pancreatic tissues in IMD-exposed tilapia; however, the hepatic steatosis persisted around the pancreocytes (Fig. 7d).

## Discussion

Exposure to 1.5 µg IMD L^−1^ significantly decreased WF and TLF, reflected in WG and TLG gain, SGR, and PER than control. These results might be due to reductions in feed intake (Günal et al. 2019). The reduction in feed intake could be assumed in the present study, whereas CCK is significantly promoted in the IMD group. This hormone suppresses stomach emptying (Le et al. 2019) and diminishes food intake (Zhang et al. 2017), via influencing stress neuroendocrine axis, thus reducing growth performance (Conde-Sieira et al. 2018; Soengas et al. 2018). Moreover, low digestive enzyme activities (amylase, lipase, and protease) and intestinal pathology in the IMD group suggested that IMD negatively influences the tilapia’s digestive system, which could contribute to delayed growth.

Administering dietary GSE®^®^ either alone or in conjunction with IMD enhanced Nile tilapia’s growth performance and feed utilization. This manifested by decreased CCK levels via GSE^®^ antioxidant potential, which mitigated IMD-induced stress. Additionally, the elevated amylase, lipase, and protease activities, combined with improved intestinal villi length and width, may result in better growth.

The IMD-contaminated water negatively affects total protein, albumin, and globulin levels. Similar results were previously documented in the study by Banaee et al. (2024), where common carp exhibited lower levels of total protein, albumin, and globulin after exposure to 10 and 20 μg/L of IMD for 28 days. The reduction in serum proteins denotes significant perturbations in fish osmotic function, immune response, and humoral protection (Jha et al. 2007; Abbasi et al. 2022). In the current study, 2% GSE^®^ raised all the protein profile levels in *O. niloticus,* parallel to Mehrinakhi et al. (2020) research on common carp using 30 g/kg GSE for 56 days.

After 75 days of IMD exposure, the MDA levels in intestinal tissues changed significantly, while CAT levels decreased. Insecticidal IMD induces oxidative stress and inflammation, accumulating free radicals in cells by disrupting the cellular antioxidant system (Sule et al. 2022); this is indicated by the increased MDA levels and reduced CAT activity. This result aligns with the findings of Abdel-Tawwab et al. (2021a) and Ramírez-Coronel et al. (2023). The GSE^®^ co-administration with IMD exhibited elevated CAT activity and reduced MDA levels, consistent with the findings of Mohammadi et al. (2021a), who demonstrated that dietary GSE at a low level enhanced CAT antioxidant defense and lowered MDA levels in hepatic tissues of carp that may be attributed to its polyphenols content that have strong antioxidant properties.

Exposure of *O. niloticus* to IMD for 75 days increased thyroid hormone levels (T3 and T4). The former increments appeared to be a physiological compensation to the IMD exposure–induced stress that could generate cortisol and ACTH, which promote thyroid hormone production peripherally (Geven et al. 2009). The elevated thyroid hormones physiologically mitigate the stressful condition via the promotion of energy expenditure and mobilization of fat to provide the fuel that causes the elevation of triglycerides (Subhash Peter et al. 2009), which further favors catabolic processes and oxidative stress (Peter and Oommen 1989), as noted in our study. The addition of 2% GSE^®^ ameliorated the increased T3 and T4 in IMD-exposed fish, enhanced growth performance, and restored the catabolic effects of the supraphysiological levels of thyroid hormones via the antioxidant potential of GSE^®^, which alleviates oxidative stress induction to cortisol (Khalifa et al. 2022).

Fish growth is a paramount concern in aquaculture, largely regulated by pivotal hormones, GH/IGF-1 and IGF-1 (Zhang et al. 2022). This research found IMD to reduce growth performance by decreasing IGF-1 secretion. However, the addition of 2% GSE^®^ improved this condition. The increased MDA and depleted CAT by IMD could promote cortisol production, which negatively influences the hepatic expression and serum levels of IGF-1 (Reindl and Sheridan 2012). A feeding diet supplemented with 2% GSE^®^ could provide an antioxidant effect (Khalifa et al. 2022), ameliorating stress and cortisol-mediated IGF-1 reduction.

The composition of the fish body, particularly protein and lipids, was used as a bioindicator in fish nutrition research and health assessments (Łuczyńska and Paszczyk 2019). Body composition is crucial in fish farming because it affects fish growth, survival, and the efficiency of food usage (Breck 2014). The body composition analysis of fish in the current study revealed that 1.5 µg/L IMD exposure decreased the whole protein contents of tilapia and increased the moisture, ash, and lipid contents. These changes might be attributed to the ability of IMD to induce lipid peroxidation and the decrease in feed intake, resulting in lower fish weights and SGR, as noted in our results. Adding 2% GSE^®^ to the diet increased the whole protein content of *O. niloticus* and decreased the moisture, ash, and lipid contents. This may be attributed to the increased feed intake, which results in high fish weights and SGR (Wang et al. 2024), as recorded in our study.

The present study indicated that IMD led to histological changes in the stomach’s glandular and fundic regions, along with a reduction in the length of the mucosal layer. IMD’s histopathological impacts on Nile tilapia stomach tissues have not been studied. Comparable stomach alterations were obtained from *C. gariepinus* fish after exposure to the quinclorac and bensulfuron-methyl pesticides for 15 days (Mohamed et al. 2023). Generally, IMD can generate oxidative stress, which may lead to changes in tissue structure and function (Abd El-Hameed et al. 2021a).

In the current histological study, sections of tilapia intoxicated with IMD displayed significant histopathological lesions in the intestinal villi, characterized by decreased length and width. A previous study by Akbulut and Ertuğ (2019) demonstrated that Zebrafish (*Danio rerio*) exposed to IMD concentrations of 9.5, 19, and 38 mg/L for 5 days exhibited similar intestinal alterations. According to the fundamental roles of the intestine in the digestion of nutrients and absorption (Fadl et al. 2021), these intestinal abnormalities cause poor digestion and absorption of essential nutritional components such as protein, lipids, and carbohydrates, thus significantly impairing fish’s growth performance and indices and impairing fish body composition. Exposure to various pesticides can harm fish tissues, leading to oxidative stress damage. This is due to the production of ROS and free radicals, which can damage the normal cellular structure of different tissues and cause histopathological injuries (Sepici-Dinçel et al. 2009), supported by the elevation of MDA levels and low CAT activities of IMD-intoxicated tilapia.

Nile tilapia given GSE^®^ showed normal mucosal tissues as compared to the control. Including dietary GSE^®^ in the diet of IMD-exposed fish in this investigation greatly reduced the digestive tissue alterations, increased the length of the mucosal digestive layer of glandular regions, and increased intestinal villi length and width as reflected by the increased digestive enzymes and growth. These effects led to improvement of the intestinal surface for nutrient absorption (Zhu et al. 2012; Fadl et al. 2021). In the same line with our findings, Mousavi et al. (2021) reported that dietary GSE at a concentration of 100 mg kg^−1^ caused growth modulation and improvement of mucosal tissues along the gastrointestinal tract of rainbow trout (*Oncorhynchus mykiss*) fry, thus indicating better defense against exposure to lethal chemicals such as IMD due to its antioxidant and antiapoptotic effects (Gao et al. 2014; Ulusoy et al. 2014).

Exposure to IMD caused degenerations in both hepatic and pancreatic tissues besides macro-steatosis or hepatocyte vacuolization. The former alteration refers to the excessive accumulation of lipids in the cytoplasm, as confirmed by Günal et al. (2019). These findings confirmed the lower levels of total protein albumin and globulin, as well as higher levels of cholesterol and triglycerides, in the sera of the IMD group. The observation of cytoplasmic vacuolations in hepatocytes indicates their degeneration, which is a common response to toxicant exposure in fish (Günal et al. 2019), where the liver has multiple roles in the metabolism and excretion of toxic xenobiotics (Omar et al. 2015). Similar alterations were pronounced in different fish species exposed to IMD: *Labeo rohita* (Qadir and Iqbal 2016), *Cyprinus carpio* (Özdemir et al. 2018), and *O. niloticus* (Günal et al. 2019). Supplementing GSE^®^ improved the appearance of hepatic and pancreatic tissues in IMD-exposed tilapia and reduced the appearance of macro-steatosis. These improvements were evidenced by lower levels of cholesterol and triglycerides and upregulation of lipase enzymes in the IMD-supplemented group than IMD group. Hence, the present findings validate the protective role of GSE^®^ against IMD-induced liver injuries. Prior studies indicate that GSE influences hepatic lipid metabolism by promoting fatty acid β-oxidation, enhancing triglycerides catabolism, and reducing fatty acid synthesis (Lu et al. 2020; Qadir and Iqbal 2016).

## Conclusions

The study concludes that feeding *O. niloticus* with 2% GSE^®^ could modulate the detrimental impacts assessed by IMD exposure. Dietary GSE^®^ enhanced the fish growth performance and the health status of IMD-intoxicated fish by regulating their physiological status, lipid profile, digestive enzymes, and growth-related hormones, along with upregulation of CAT activities and lowering MDA levels in intestinal tissues. Moreover, it enhances the chemical composition of the fish’s body and mitigates the adverse histological changes induced by IMD in the stomach, intestine, and hepatopancreatic organs. As a result, this study holds significant implications for Nile tilapia, given its economic importance and its impact on the quality and safety of human consumption. The study only examined a single concentration of IMD (1.5 µg L^−1^) and GSE (200 mg kg^−1^) in diet without evaluating dose–response relationships, which are essential for determining toxicity thresholds and optimal GSE dosages. A limitation of the study is that while GSE supplementation offers long-term protection, it remains unclear if continuous exposure to IMD could diminish its benefits. The study lacks generalizability across different species and does not account for environmental variability. This highlights the need for further research on the role of GSE in reducing IMD toxicity, as well as its ecological implications and cost-effectiveness in commercial aquaculture. Additionally, potential environmental consequences, such as bioaccumulation and changes to microbial ecosystems, have not been addressed. Future studies should focus on the stability and degradation of GSE in feed and its effects on the aquaculture ecosystem. It is also recommended to explore hormonal interactions, dose–response relationships, and the feasibility of GSE supplementation in practical applications.

## Supplementary Information

Below is the link to the electronic supplementary material.

Supplementary file1 (DOCX 670 KB)

Supplementary file2 (DOCX 18 KB)

## Data Availability

No datasets were generated or analysed during the current study.
