# Supplementary material for: Dietary grape seed extract mitigated growth retardation, hormonal delay, and gastrointestinal toxicity induced by insecticide imidacloprid in Nile tilapia
Source: Fish Physiol Biochem. 2025 Mar 25;51(2):72. doi: 10.1007/s10695-025-01475-1 (PMC11937218; doi:10.1007/s10695-025-01475-1)
Supplement: Supplementary file 1 — Supplementary file1 (DOCX 670 KB) [file 10695_2025_1475_MOESM1_ESM.docx]

**
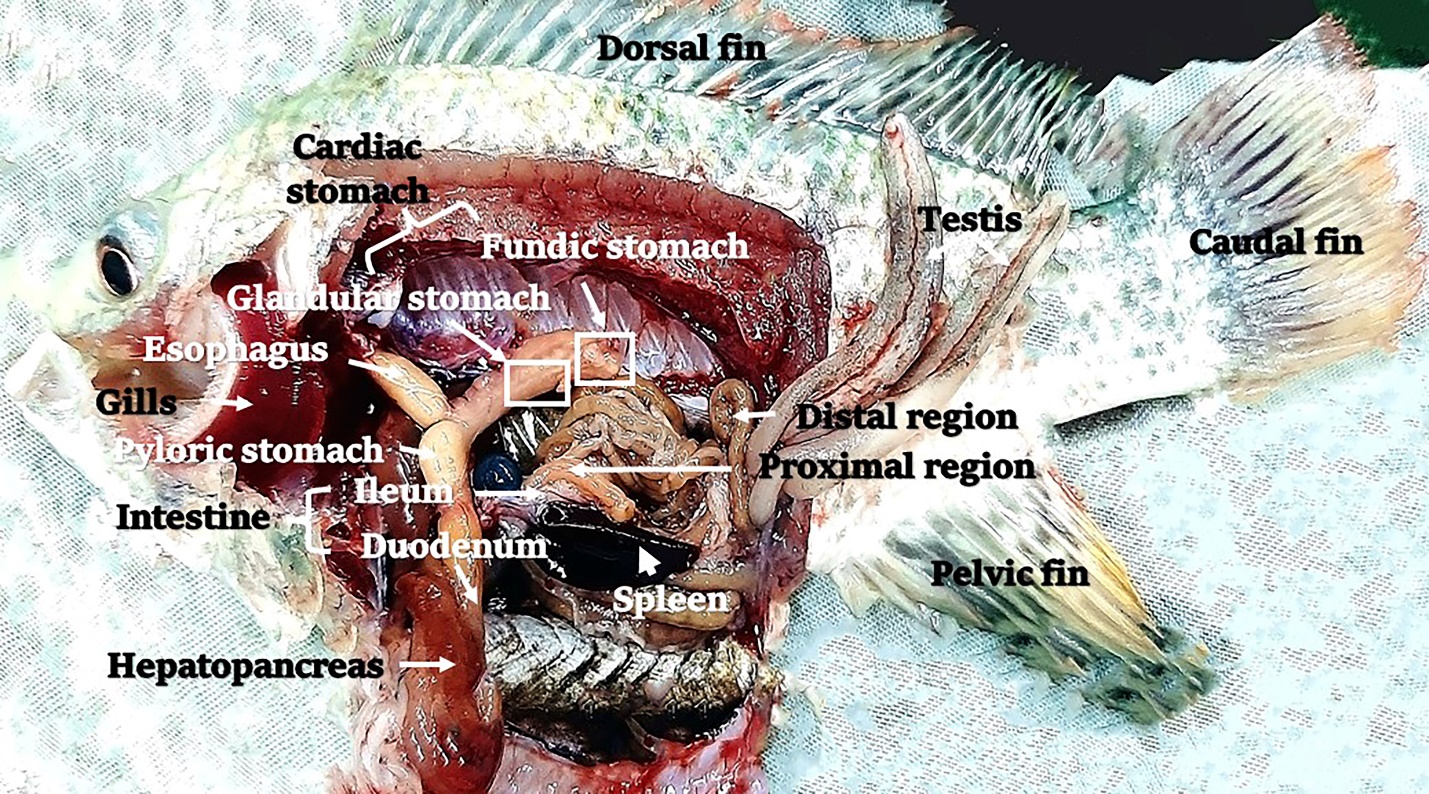
**

**Fig S1:** Macroscopical organization of the digestive tract in Nile tilapia (*Oreochromis niloticus*).
