# Supplementary material for: Dietary grape seed extract mitigated growth retardation, hormonal delay, and gastrointestinal toxicity induced by insecticide imidacloprid in Nile tilapia
Source: Fish Physiol Biochem. 2025 Mar 25;51(2):72. doi: 10.1007/s10695-025-01475-1 (PMC11937218; doi:10.1007/s10695-025-01475-1)
Supplement: Supplementary file 2 — Supplementary file2 (DOCX 18 KB) [file 10695_2025_1475_MOESM2_ESM.docx]

**Table S1:** Experimental design continued for 75 days.

| **Experimental group**  **(60 fish/group)** | **Standard diet** | **2 % GSE^®^** | **1.5 µg IMD L^-1^** |
| --- | --- | --- | --- |
| **Group 1 - Control** | **√** | **-** | **-** |
| **Group 2 - GSE^®^** | **-** | **√** | **-** |
| **Group 3 - IMD** | **√** | **-** | **√** |
| **Group 4 - GSE^®^**  **+ IMD** | **-** | **√** | **√** |

GSE**^®^**: grape seed extract, IMD: imidacloprid.

| **Ingredient** | **Standard diet**  **% GSE ^®^** | **2 % GSE ^®^** |
| --- | --- | --- |
| **Soybean meal (48%)** | **18 %** | **18 %** |
| **Fish meal (70%)** | **21 %** | **21 %** |
| **Wheat bran (15%)** | **48%** | **48%** |
| **Corn gluten meal (60****%)** | **11 %** | **11 %** |
| **Corn oil** | **1 %** | **1 %** |
| **Vitamin and mineral mix * (0.5%)** | **0.5 %** | **0.5 %** |
| **Salt** | **0.5 %** | **0.5 %** |
| **GSE^®^ (13.1%)** | **-----** | **2%** |
| **Biochemical status of experimental diets (%)** | | |
| **Dry matter (%)** | **89.67** | **89.76** |
| **Crude protein (%)** | **37.75** | **37.05** |
| **Crude fat (%)** | **6.06** | **6.06** |
| **Crude fiber (%)** | **1.61** | **1.61** |
| **Gross energy (MJ\Kg)** | **19.65** | **19.54** |
| **Moisture (%)** | **10.33** | **10.33** |

**Table S2:** Compositions of standard and supplemented diets of Nile tilapia

* Each Kg vitamin & mineral mixture premix contained the following ingredient; 4800 IU Vit A, 2400 IU cholecalciferol (Vit D), 40g Vit E, 8g Vit K, 4g Vit B12, 4g Vit B2, 6g Vit B6, 4g pantothenic acid, 8g nicotinic acid, 400mg folic acid, 20mg biotin, 200mg choline, 4g copper, 0.4g Iodine, 12g Iron, 22g manganese, 22g zinc, 0.04g selenium folic acid, 1.2mg niacin, 12mg d-calcium pantothenate, 26mg pyridoxine HCl, 6mg riboflavin, 7.2mg thiamin HCl, 1.2mg sodium chloride (NaCl, 39% Na and 61% Cl), 3077mg ferrous sulfate (FeSO4.7H2O, 20% Fe), 65mg manganese sulfate (MnSO4, 36 % Mn), 89 mg zinc sulfate (ZnSO4.7H2O, 40 % Zn), 150mg copper sulfate (CuSO4.5H2O, 25 % Cu) and 28mg potassium iodide (KI, 24 % K and 76 % I).
